# Disordered eating and considerations for the transgender community: a review of the literature and clinical guidance for assessment and treatment

**DOI:** 10.1186/s40337-023-00793-0

**Published:** 2023-05-15

**Authors:** Kerry McGregor, John L. McKenna, Ellis P. Barrera, Coleen R. Williams, Sydney M. Hartman-Munick, Carly E. Guss

**Affiliations:** 1 Gender Multispecialty Service, Boston Children’s Hospital, Boston, MA USA; 2 Division of Adolescent and Young Adult Medicine, Boston Children’s Hospital, Boston, MA USA; 3 Department of Psychiatry, Harvard Medical School, Boston, MA USA; 4 Division of Pediatrics, Harvard Medical School, Boston, MA USA; 5 UMass Memorial Children’s Medical Center/UMass Chan Medical School, Worcester, MA USA

**Keywords:** Eating disorders, Disordered eating behaviors, Transgender, Gender diversity, LGBTQ

## Abstract

**Background:**

It has been well established that individuals who identify as lesbian, gay, bisexual, transgender, and queer are at increased risk for mental health pathology, including eating disorders/disordered eating behaviors (ED/DEB). However, less is understood about the unique experiences of transgender and gender diverse (TGD) people who struggle with ED/DEB.

**Aims:**

The purpose of this literature review is to examine the literature regarding the unique risk factors for TGD individuals who experience ED/DEB through a lens informed by the minority stress model. Additionally, guidance around the assessment and clinical management of eating disorders for TGD individuals will be presented.

**Results:**

TGD people are at increased risk for developing ED/DEB due to a number of factors including: gender dysphoria, minority stress, the desire to pass, and barriers to gender affirming care.

**Conclusion:**

While guidance around assessment and treatment of ED/DEB for TGD individuals is still limited, adhering to a gender affirmative care model is essential.

## Abstract (summary)

People who identify as lesbian, gay, bisexual, transgender, and queer (LGBTQ) are more likely to have mental health difficulties, including eating disorders/disordered eating behaviors (ED/DEB). Much of the existing literature has focused on LGBTQ populations and not on the unique experiences of transgender and gender diverse (TGD) people. This literature review seeks to examine the unique risk factors for TGD people who experience ED/DEB. The minority stress model is used to better understand these risk factors, which include gender dysphoria, minority stress, the desire to pass, and barriers to gender affirming care. Research offering guidance for assessing and treating ED/DEB with TGD people is still limited but it is clear that a gender affirmative care model provides robust support for this population.

## Background

Eating disorders and disordered eating behaviors (ED/DEB) have long been associated with poorer quality of life, potentially lifelong physical impairments, and higher rates of mortality [1]. Although ED/DEB affect people of many different racial identities, ethnic backgrounds, and sexual identities, studies have highlighted that transgender and gender diverse (TGD) populations are especially at risk [2–5]. Historically, research regarding disordered eating has not represented the unique experiences of TGD people and emerging research that seeks to do this is underfunded, which unfortunately has limited scholarly progress in developing clinical knowledge of how to best identify and help TGD individuals struggling with ED/DEB [6–8].

Despite this research domain still being in its infancy, scholars and clinicians have begun to explore factors that may lead to heightened risk for eating disorder pathology for TGD people. There is also a body of literature that guides clinical best practices for assisting TGD people with mental health challenges using an affirming and responsive approach. The purpose of this literature review is to synthesize these areas of study to help guide assessment and management of ED/DEB for TGD people. Specifically, this literature review aims to (a) offer an overview of important terminology related to gender and the TGD community, (b) use a minority stress framework to explore contributing factors to ED/DEB development among TGD people, (c) provide an overview of available eating disorder screeners and clinical assessments, and (d) present current treatment recommendations to best assist providers in effectively treating TGD individuals who struggle with disordered eating.

## Transgender and gender diverse identities

Approximately 1.4 million adults and 150,000 youth between the ages of 13 and 17 identify as transgender in the United States [9, 10]. The phrase transgender and gender diverse (TGD) is often used as an umbrella term to describe individuals who have a gender identity that is different than their assigned sex at birth (ASAB). This is in contrast to a cisgender identity, which indicates that one’s ASAB aligns with one’s gender identity (e.g., an individual assigned male at birth goes on to identify as a boy/man, an individual assigned female sex at birth goes on to identify as a girl/woman) [11].

It is important to note that gender is a complex and multifaceted construct that extends beyond the gender binary, or the assumption that only two genders exist. Gender identity describes how one defines and labels their own gender; it is an internally held construct. One’s gender identity can therefore be completely separate from gender expression—or the outward appearance or presentation of gender that is interpreted by others based on gender norms [12]. An example of this would be a TGD person who identifies as transmasculine while their gender expression is considered to be more conventionally feminine (e.g., wearing dresses, painting their nails, and wearing makeup). The constructs of gender identity and expression have been conceptualized as existing on masculine and feminine spectrums, while also allowing individuals to reject gender labels and a binary understanding of gender altogether [13]. To assist in understanding different gender identity labels and components of gender, Table 1 includes a summary of common terms and definitions to assist clinicians and healthcare professions.

**Table 1:** Important terms to know when best serving a TGD population [11]

Term	Description
Gender identity	An inner sense of one’s own gender
Gender expression	The outward appearance or manifestation of gender, such as clothing, hair, voice infliction, and mannerisms
Gender affirming care	Healthcare that is designed to support and affirm an individual’s gender identity
Assigned sex at birth	Classification of a person as male, female, intersex, or another sex based on anatomy (such as genitalia) and chromosomes
Transgender	An umbrella term for people whose gender identity is different that associated with their assigned sex at birth
Cisgender	A term to describe someone whose gender identity corresponds with their assigned sex at birth
Non-binary	An umbrella term that encompasses gender identities that do not fit within the gender binary (e.g. binary that defines gender as male/female only). People who use this term to describe their gender identity tend to reject the notion that gender must be dichotomous (man/male/masculine vs. woman/female/feminine) and based on sex assigned at birth. Words that people may use to describe their non-binary identity include “gender-fluid”, “gender nonconforming”, and “genderqueer.”
Transmasculine	An umbrella term that indicates a person’s gender identity is more masculine. This term is most often used by people assigned female sex at birth who affirm a masculine-leaning gender
Transfeminine	An umbrella term that indicates a person’s gender identity is more feminine. This term is most often used by people assigned male sex at birth who affirm a feminine-leaning gender
Agender	An identity where the individual does not see themselves as having a gender or a gender identity (this can fall under a gender-void umbrella). This is separate from non-binary identities, which implies a gender identity that may be a combination of masculinity and femininity
Cisnormativity	The inherent societal assumption that being cisgender is “normal” or “correct”, and that people with other genders should strive to be cisgender. This ideology often leads to the marginalization and discrimination of transgender and gender diverse individuals
Transphobia	A fear, dislike of, or prejudice against TGD people
Internalized transphobia	The inward direction of transphobia to the self. This can result in some TGD people feeling ashamed or self-hatred for their gender diversity and may seek to hide their gender from others. Some people prefer the term “internalized transnegativity” because this locates prejudice within society rather than within the individual

Meanings associated with terms are rapidly evolving and led by TGD community members and activists. The included descriptions in this table are considered to represent common definitions at time of publication but it is strongly encouraged that providers continue to self-educate to be aware of up-to-date terminology

## Minority stress model

In order to best understand ED/DEB among TGD individuals, it is important to understand how minority stress contributes to the development of physical and mental health challenges. A minority stress model posits that marginalized communities experience greater instances of mental health challenges because such populations face additional life stressors due to having to navigate oppressive and hostile social contexts [14]. Meyer conceptualized dimensions of minority stress from cisgender gay, lesbian, and bisexual (GLB) people as existing on a continuum ranging from distal to proximal stressors [15, 16]. Distal stressors are external events that are directed toward an individual, such as acts of harassment, violence, and victimization; this is typically understood to be rooted in transphobia, homophobia, and other systemic prejudices and discriminatory practices. Proximal stressors are an individual’s appraisals or subjective responses to the event, which is a distal stressor. This may include hypervigilance, anticipating stigmatization by others, hiding or rejecting one’s identity, or internalizing negative societal attitudes and prejudices [16].

Hendricks and colleagues were one of the first to adapt this model to account for stress among TGD individuals. These researchers noted that TGD people face tremendous structural stigma (e.g. restriction of equal opportunities for those with mental health challenges) and inequities rooted in oppression of TGD identities and experiences [17, 18]. It is believed that gender socialization that begins in childhood reinforces a gender binary and establishes a range of socially-acceptable displays of masculinity for boys and femininity for girls. Children or individuals that do not fit within this binary are at substantial risk for distal stressors (i.e., transphobia, violence, etc.) due to society’s reaction to their gender diversity in identity and expression (19).

Understandably, TGD individuals come to expect and prepare to be emotionally and/or physically harmed by others and may feel motivated to hide or fully reject their own gender diversity. Consequently, TGD people might then develop internalized transphobia, which can be explained as a process of directing negative societal attitudes about gender diversity towards themselves [20]. According to a minority stress model, internalized transphobia is a significant proximal stressor and is characterized by self-blame and low self-esteem that reinforces a cycle of negative appraisal of the self for being transgender [17]. This then can lead to a poor self-image, which negatively affects self-worth, self-regulation, and body image. These factors have been posited to be a major contributor to TGD individuals developing disordered eating habits [21].

## TGD people and disordered eating

To date, prevalence rates of ED/DEB among TGD communities are estimated between 2 and 18% according to a scoping review including 20 publications [22]. One study with a sample size of 1,333 TGD youth found that approximately 4.3% of transmasculine and 4.2% of transfeminine youth reported a lifetime eating disorder diagnosis [23]. Another study found that approximately 18% of trans individuals reported an eating disorder diagnosis in the past year compared to 1.8% of cisgender female youth and 0.2% of cisgender male youth [24]. A more recent review by Nagata et al. estimated TGD people in the United States to have a lifetime prevalence of diagnosed disordered eating at a rate of 10.5% for transgender men and 8.1% for transgender women. Of note, the most common diagnoses were anorexia nervosa (4.2% for transgender men and 4.1% for transgender women) and bulimia nervosa (3.2% for transgender men and 2.9% for transgender women) [25]. Such significantly heightened prevalence rates signal a critical need to better understand what contributes to the onset and development of ED/DEB for this population. To date, the extant body of literature has identified a handful of potential contributing factors for the development ED/DEB pathology, which include: (1) puberty and gender dysphoria; (2) cisnormativity and passing; and (3) barriers to accessing gender affirming healthcare.

### Gender dysphoria

TGD people may experience gender dysphoria – a significant level of psychological distress that is caused by an incongruence between one’s gender identity and their sex assigned a birth [26]. This misalignment between one’s gender identity and their primary and secondary sex characteristics is often associated with a high level of dissatisfaction with one’s body and general appearance [27–29]. For many TGD individuals, disordered eating can be understood as a method of “either suppressing or accentuating gender by changing the shapes of their bodies” (pg. 73) [30].

Given that some TGD individuals may feel their gender dysphoria decrease as a result of restrictive eating and accompanying body changes, there is significant reinforcement around the development and maintenance ED/DEBs. For individuals who underwent an estrogen-driven puberty but affirm a masculine or male gender identity, ED/DEB may be motivated by a desire to present more conventionally masculine. Specifically, weight loss through restrictive eating can reduce the size of breasts/chest area, hips, and buttocks, in addition to causing amenorrhea [8]. Among individuals who underwent a testosterone-driven puberty but affirm a feminine or female gender identity, weight loss may help reduce broadness of shoulders and create the appearance of a smaller body frame that may be considered more conventionally feminine [30, 31].

### Passing and scrutiny of TGD bodies

When a TGD individual presenting as their affirmed gender identity is perceived as cisgender by others, or is not suspected to be transgender, then that person is considered to successfully “pass” [31, 32]. For some TGD people, it is an important goal to be seen and understood by those around them as a cisgender individual for a variety of reasons, such as safety concerns and a sense of belonging [33, 34]. TGD people who do not “pass” or whose gender expression is outside the gender binary may be susceptible to more transphobic discrimination, harassment, and violence [35]. As a result, TGD individuals with a binary gender identity (e.g., transgender men, transgender women) may feel tremendous pressure to adhere to social expectations of traditional gender roles and gender expression that cisgender men and women adhere to in order to garner as sense of safety in their communities [29]. It is important to note that “passing” is not a goal for all TGD people, even with binary gender identities; each TGD person is unique and healthcare clinicians should not assume that “passing” as cisgender is a desired goal or outcome.

Although the relationship between “passing” and ED/DEB is understudied, researchers have noted that the impact of “visible” gender-nonconformity can lead some TGD people to turn to ED/DEB in response to the minority stress of having to exist in a cisnormative society. Disordered eating can therefore be understood as a strategy that is employed to control one’s body with the hopes of “passing” and/or can be the result of stress and anxiety from feeling as though “passing” is ultimately unattainable [31, 36]. From this perspective, ED/DEB can therefore be conceptualized as a resulting psychopathology that is secondary to the significant levels of distress that TGD people expereince from being subjected to a transphobic cultural context, which, in of itself, can be considered to be a primary risk factor [37]. Scholars have also commented that narrow beauty standards for conventional masculinity and femininity are significantly amplified for TGD individuals [38, 39]. Chang and colleagues noted that TGD people face more scrutiny about their bodies “because they are expected to ‘prove’ themselves as being ‘man enough,’ ‘woman enough,’ or ‘trans enough’” (p. 116) [40]. Additionally, some non-binary people might feel pressured to maintain a solely androgynous physical appearance so that others cannot discern assigned sex at birth [41]. Regardless, this hyper-scrutiny of bodies that perpetuates toxic beauty standards that is often reinforced by social media particularly for TGD youth, likely magnifies TGD people’s negative views of their bodies and can contribute to ED/DEB [42].

### Barriers to affirming care and disempowerment

Access to gender affirming care is considered to be essential to the wellbeing of TGD people. Such healthcare can include having the option to pursue affirming medical interventions, such as hormones, pubertal suppression, and various surgeries, as well as receiving care from healthcare providers that are knowledgeable about TGD identities and relevant medical implications [43]. Unfortunately, research has repeatedly shown that TGD individuals encounter discrimination when attempting to access general mental and medical healthcare and that their needs are often left unmet [43–45]. Mistreatment within a medical context can take many forms, including misgendering (i.e., referring to a patient using incorrect pronouns) or misnaming (i.e., using the incorrect name), in addition to outright refusal of services [46]. TGD people may also not be able access affirming services due to geographic region, limited support from family or caregivers, financial limitations, and long waitlists for care [47].

These barriers to and negative experiences accessing gender affirming care can contribute to TGD people engaging in ED/DEBs. Malina hypothesized that some TGD people engage in disordered eating to gain a sense of power in response to being deprived of medically necessary and appropriate care [31]. Kosciewicz and colleagues similarly assert that, because TGD people are disempowered in the current healthcare system, this population may turn to disordered eating in an attempt to gain some sense of control over one’s body since access to adequate treatment for mental health challenges and dysphoria is not possible [30]. Therefore, weight loss via ED/DEB can be viewed as the only viable option for some TGD people to help themselves feel better or more empowered.

### Protective factors

Although there are multiple factors that can contribute to the development and maintenance of ED/DEB among TGD people, research has also identified multiple protective factors that promote wellbeing for this resilient community. For example, studies have found that access to gender-affirming medical interventions may reduce risk of ED/DEB for TGD communities [8, 46, 48]. Additionally, findings from one study indicated that family connectedness, school connectedness, caring friends, and social support were linked to lower odds of disordered eating using a sample of 925 transgender young adults [49]. Social support and connectedness have also been found to be a critical protective factor for TGD people against many other mental health challenges, such as depression and anxiety [50]. Better understanding and promotion of these protective factors for TGD people struggling with ED/DEB will aid in clinicians’ efforts to promote resiliency in this population over the long term.

## Screening and assessment of disordered eating among TGD people

Within the past five years, there has also been a call for clinicians to find ways to screen for eating disorder symptoms more appropriately and accurately within the TGD population to gain a better understanding of psychological functioning and to guide treatment recommendations. Unfortunately, many historical gold-standard screeners for disordered eating—such as the Eating Disorder Inventory (EDI)—have been developed solely for cisgender people [51–54]. Therefore, such measures may contain items or subscales that do not properly assess disordered eating attitudes or behaviors for TGD people. Gordon et al. noted that questions from the EDI in particular may hold different meanings for TGD individuals. For example, the item “I feel satisfied with my body image” may reflect gender dysphoria and the item “I wish I was someone else” might unintentionally capture experiences of transphobia instead of disordered eating alone [36, 53].

Thankfully, there has been an increase in research examining eating disorders in TGD populations in recent years, which has contributed to the use and validation of eating disorder screeners with TGD individuals. Table 2 includes an overview and relevant findings with TGD samples of the Eating Disorder Examination Questionnaire (EDE-Q), the short form of that questionnaire (EDE-QS), the Eating Attitudes Test (EAT-26), and the Sick, Control, One Stone, Fat, Food (SCOFF) questionnaire [51, 55–57].

**Table 2:** Quantitative assessments of ED/DEB used with TGD populations

Name	References	Items	Assessment	Statistics with TGD populations
Eating Disorder Examination Questionnaire (EDE-Q)	Fairburn and Beglin [55]	28	Assesses eating disorder attitudes and disordered eating behaviors over the past 28 days; four subscales: restraint, eating concern, shape concern, weight concern	Adequate to excellent internal consistency with transmasculine, transfeminine, and nonbinary youth and adult transgender men and women [25, 58]; a single-factor global score may be optimal for TGD youth [59]
Eating Disorder Examination Questionnaire—Short Form (EDE-QS)	Giedon et al. [60]	12	Same as EDE-Q; yields single score	Strong measurement and construct validity for transgender men and women with and without suspected ED/DEB [56]
Eating Attitudes Test (EAT-26)	Garner and Garfinkel [51]	26	Indicates to respondent if they have symptoms of an eating disorder that warrants professional attention; yields single score with score > 20 indicating further investigation by a clinician	Lower scores on EAT-26 associated with more experience with gender-affirming medical interventions [8]; good to excellent internal consistency with TGD young adults and adult transgender women [61, 62]
Sick, Control, One Stone, Fat, Food (SCOFF)	Morgan et al. [57]	5	Identifies individuals with disordered eating in a primary care context; response of “yes” to at least two questions indicate more in-depth assessment of anorexia and/or bulimia is required	Has been used in a case series examining gender-affirming nutrition with adult transgender men, a cross-sectional nutrition screening protocol with transgender and nonbinary youth, and the validation of an avoidant/restrictive food intake disorder measure with transgender and nonbinary youth [63–65]

*Qualitative inquiries* Although quantitative screeners offer specific cut-off scores that carry clinical utility, it is important not to undermine the value of using open-ended questions as well. Donaldson and colleagues used qualitative data from five case studies of adolescent TGD youth with eating disorders to identify common features and guide eating disorder screener questions for both providers in a gender clinic, and providers in an eating disorder clinic [66]. Recommended questions to ask TGD youth in an eating disorder clinic included asking about (a) gender identity, (b) how a patient feels about their body in relationship to their gender, and (c) what goals the patient has for their body. Providers assessing disordered eating in a gender clinic were recommended to ask questions related to: (a) disordered eating in general, (b) what the patient has eaten and drank in the last 24 h, (c) their relationship to physical activity and exercise, (d) their history of dieting or compensatory behaviors, and (e) how they feel about their body in general [66]. These types of questions allow for a deeper discussion with TGD youth about their relationship to their bodies and food, and how gender dysphoria may play an important role.

In sum, the recent increase in research examining the experiences of disordered eating among TGD people has led to the application of existing eating disorder screeners and the creation of specific questions that clinicians can ask to better assess ED/DEB for people who may also experience gender dysphoria. The incorporation of screeners during work with TGD people can assist clinicians in properly identifying which patients could benefit from targeted, affirming interventions specifically related to disordered eating.

## Clinical practice implications

To date, there are no consensus guidelines for the treatment of ED/DEB among TGD individuals and studies examining clinical care outcomes for this population are limited. TGD patients may present to care more medically compromised than cisgender individuals [67, 68]. However, a literature search of peer reviewed work showed that some studies have captured the overwhelmingly negative experiences that TGD people have had when undergoing ED/DEB screening and treatment. For instance, in a survey of 84 TGD respondents who had completed some form of ED/DEB treatment, none reported having a positive experience [69]. Approximately 40% of respondents hid their gender identity from their treatment program out of fear of discrimination and stigma, and those who did disclose their identity faced misgendering, transphobia, and various non-affirming questions from providers [69]. Another study found that TGD patients who sought treatment for ED/DEB were frustrated by the limited number of providers who were both knowledgeable about gender affirming care and had expertise in addressing disordered eating [70]. Given these findings, it is important for clinicians to understand what constitutes gender affirming care.

### Affirmative care

According to the World Health Organization, gender affirming care encompasses psychological, behavioral, and medical interventions that are “designed to support and affirm an individual’s gender identity” when that identity does not align with sex assigned at birth [71]. A core philosophy of gender affirming care is that variations in gender identity and expression are natural, expected, and not pathological [72]. Therefore, when providing care to TGD people, an affirmative approach includes valuing patient autonomy and the idea that patients are experts of themselves, and that patient identities are deserving of acknowledgement and respect. In practice, gender affirming care, at minimum, includes always using the patient’s correct name and pronouns both in-and-out of the exam room, asking questions about the body that are not inherently gendered (e.g., saying “chest” instead of “breasts”, etc.), and having resources for TGD patients when their questions or care needs are outside the scope of a clinician’s expertise. Providing care within a space that indicates inclusivity and affirmation with observable signs (e.g., pride flag lanyards, rainbow stickers, etc.) can also be of assistance considering that TGD people are more likely to scan their environment for signs of safety [73, 74].

### Affirming care for TGD people with ED/DEB

When applying gender affirming care principles to treating ED/DEB, it is important for providers to acknowledge that the very nature of “recovery” from disordered eating for TGD people may exacerbate the gender dysphoria they experience [75, 76]. For example, a young transgender man assigned female at birth recovering from anorexia nervosa may experience heightened dysphoria because of weight gain and non-affirming changes to the body like re-emergence of body curves, more chest development, and return of menses [77, 78]. Therefore, clinicians working with this population should become familiar with ways of helping TGD patients find other avenues of addressing gender dysphoria that does not include diet or exercise. This can include finding gender euphoria, the joy or happiness from one’s gender identity and expression, through other avenues that can include but are not limited to hair styles, clothing, and makeup [79]. There are also gender affirming medical interventions, such as pubertal blockade, hormones, and surgeries that can be exceptionally helpful in reducing dysphoria for TGD individuals [80–82].

When conceptualizing ED/DEB etiology and manifestation for TGD individuals, it is critical to remember that some traditional but cis-normative treatment models for alleviating disordered eating may focus on helping a patient find love and acceptance of their bodies. This treatment approach may be quite invalidating for TGD people and the gender dysphoria they experience. The concept of love for one's own body can be perceived as being in direct opposition for TGD people living with dysphoria and who may have goals of wanting to medically transition to embody their affirmed gender. Malina and Koscieweicz and colleagues emphasize that TGD bodies exist within social and political contexts, and, therefore, eating disorders are neither solely situated within the TGD individual nor purely cognitive [30, 31]. Chang and colleagues recommend that the incorporation of acceptance-based and dialectic behavioral therapy strategies may be especially helpful in the treatment of ED/DEB among TGD individuals given the dual focus on acceptance of current circumstances and experiences along with the desire for change to take place [40]. Although there is a dearth of research investigating the utility of family-based interventions to assist TGD youth specifically with ED/DEB, these types of interventions have been found to be exceptionally helpful for cisgender youth [83]. Given family connectedness is considered to be a primary protective factor for TGD young people, it is important that future studies explore the effectiveness and application of this treatment approach for this vulnerable population [66].

Clinicians offering support to this population may also find it to be helpful to develop questions aimed at better understanding the presence and/or function of ED/DEB. Two example prompts include: (1) “Some people may restrict (or purge, over-exercise, binge eat, use weight loss supplements, or muscle building supplements) to appear more masculine or feminine, or to “pass.” Has that ever been true for you?” and (2) “What does the ideal body look like to you?” These questions can help lead the clinician to better understand the motivation behind some DEBs for a TGD person, therefore, may help with important insights for guiding treatment. Additional sample questions that clinicians can use to more thoroughly assess the intersection of gender identity and ED/DEB among TGD people is offered by Donaldson and colleagues [66].

## Medical considerations

From a medical assessment perspective, little guidance exists on how to best work with TGD individuals who have ED/DEB. However, based on the clinical experience of some of the authors of this paper, who are cisgender adolescent medicine physicians with a specialty in ED/DEB, there are modifications to current clinical practices that should be considered to help TGD people feel more comfortable. For example, changing into a gown can be difficult for these patients given the presence of dysphoria, and so informing patients in advance of the need for a physical exam can be helpful. Modifying what can be worn during an exam, as appropriate for medical care, can also be an affirming practice to incorporate into exams. For example, if an exam can be conducted without having the patient change into a hospital gown that may be ideal. Also, allowing gender-affirming garments to remain on, such as chest binders to flatten a person’s chest or gaffs to conceal genitals, during an exam can offer some relief from dysphoria and possibly create better engagement from the individual during a medical visit [66]. Other measures can also be taken by the medical professions to help reduce and mitigate dysphoria during appointments, such as asking what words are affirming for a patient to use with regard to the body/anatomy, discussing what a physical exam will entail prior to conducting the exam, explicitly asking for and getting consent for the exam, stopping the exam if consent is withdrawn by the patient, and limiting the duration of the exam [66, 84]. Steps such as these will help to create a safe and affirming medical exam space not only for TGD patients, but for patients of all gender identities [68, 85].

The use of growth charts for weight restoration in TGD individuals is an area that requires further study especially for those who suppress puberty and go on to medically transition with gender affirming hormones [86]. It may be helpful for physicians to consider using TGD patients’ prior weight trajectory, as well as other signs of malnutrition (e.g., heart rate, blood pressure, and laboratory evaluation) when assessing ED/DEB severity and progress with treatment [67].This patient-specific information can be especially helpful with setting realistic and achievable recovery goals for TGD people at each stage of the medical transitioning journey.

## Discussion

This literature review detailed key themes among published clinical research regarding the prevalence, screening, treatment, and experiences of TGD people with ED/DEB. While the research on the experiences of TGD people with disordered eating is limited, the research reviewed does establish that TGD individuals are at increased risk when compared to cisgender peers for ED/DEB. Much of this increased risk is explained within the minority stress model, which contextualizes the unique stressors that TGD people experience including stigma, discrimination, and prejudice (e.g., transphobia) [16, 17]. It is well-established that these stressors lead to increased rates of mental health related distress, including disordered eating, in TGD individuals [17].

Emerging research has offered more nuance that aids in a comprehensive understanding of the interplay between gender dysphoria and ED/DEB. The majority of evidence, thus far, has pointed to the inherent risk for ED/DEB created by the dissatisfaction when one’s body and appearance is not in line with their gender identity [27, 28]. TGD individuals who experience gender dysphoria can look to ED/DEB in order to cope with their distress and change the appearance of their bodies [8, 31]. This can be significantly reinforced through secondary gain when individuals potentially “pass” better as their affirmed gender and/or experience decreased dysphoria [8, 30, 31, 33, 34, 54].

Given the significant number of risks for the TGD population, it is essential that clinicians are appropriately informed on how to best assess and treat ED/DEB with this community [8, 22, 29, 39]. Many of the screeners that are widely used in clinical settings have been developed for cisgender people and may have some questions that are not easily adaptable to TGD patients [36, 53, 54]. However, as presented in Table 2, work is being done to validate these measures with TGD populations. Before using a measure to capture ED/DEB among TGD individuals, it is strongly recommended a clinical team carefully review each question and make a determination about which measure is most applicable to their patient population. Once assessment and diagnosis have been completed, clinical guidance on treatment protocols for TGD patients are limited. However, foundational aspects of gender affirming care are required as part of any ED/DEB treatment. This includes affirming patients’ identities, using the correct name and pronouns, and utilizing visual cues of inclusivity/affirmation. Consideration of a patient’s gender identity is also essential when considering bathroom access and room assignments for inpatient care [87]. Gender identity is also an important consideration for any care model that includes delivery of treatment in a group format that is separated by gender; in this case, it is recommended to discuss the options for groups based upon gender with the TGD client and allow them to participate in the group that is most affirming and authentic.

The literature reviewed has limits in generalizability, primarily due to the paucity of scholarship in this area, although this is improving with time. To date, many studies have used general LGBTQ samples rather than focusing on TGD participants and have had an overwhelming focus on restricted eating rather than binge eating, ARFID, etc. Further, TGD samples are also often compared to cisgender samples and tend to be very binary, which may not allow for nuance in understanding how the experiences of TGD people may be shaped by their affirmed gender. In addition, many studies lack diverse samples, and thus little is currently known about the impact of cultural, racial, or ethnic diversity. Many samples are predominantly white, and the specific experiences of TGD youth of color are not well understood in the current research and clinical realms. This is particularly concerning given that TGD people of color have been shown to have significantly higher risk factors healthcare discrimination [88, 89]. Future research should focus on representative samples of TGD individuals of varying gender identities, cultural experiences, and racial/ethnic backgrounds. Furthermore, research should highlight the efficacy and validity of current ED/DEB measures for TDG populations and determine what additional measures are needed.

## Conclusion

While some strides have been made to better understand identification, assessment, and treatment of ED/DEB for the TGD population (see Table 3 for a summary), it’s clear that there is a significant need for future research and clinical developments. It will be crucial that researchers and clinicians across all areas of TGD and ED/DEB care collaborate to address these areas. Centering the gender affirming approach will help to ensure best practices for working with the TGD community.

**Table 3:** Quick reference and summary of clinical recommendations

1	The TGD youth are found to be at a heightened risk for DEB/ED
2	Unfortunately, many screening measurements were developed for cisgender populations and as a result may not fully capture the experiences of TGD individuals. Recommended questions to ask TGD youth in an eating disorder clinic included asking about (a) gender identity, (b) how a patient feels about their body in relationship to their gender, and (c) what goals the patient has for their body
3	Research indicates that gender affirming hormones are associated with higher quality of life and decreased suicidality. We encourage clinicians treating TGD patients with ED/DEB to prioritize continued access to GAH during treatment/recovery
4	Gender affirmation has been associated with positive psychosocial outcomes. Additionally, TGD patients report a high level of medical discrimination. Therefore, it is important to consider the following general clinical recommendations
	(a) Avoiding misgendering/deadnaming is crucial and should be done at all points of the clinical process, from the waiting room to the exam room (b) It is important to ask questions about the body that are not inherently gendered (e.g., saying “chest” instead of “breasts”, etc.) (c) Having observable signs that a provider is affirming and LGBTQ+—inclusive—(e.g., pride flag lanyards, rainbow stickers, etc.) can also help create an environment of safety for TGD patients
5	Medical considerations include:
	(a) Modifying the physical exam to include allowing gender-affirming garments to remain on, such as chest binders to flatten a person’s chest or gaffs to conceal genitals (b) Asking what words are affirming for a patient to use with regard to body/anatomy (c) Discussing what a physical exam will entail prior to conducting the exam and explicitly asking for and getting consent for the exam. It is also important to stop the exam if consent is withdrawn by the patient (d) Use of growth charts for weight restoration in TGD individuals is an area that requires further study since growth charts may be inaccurate due to normative values being calculated from cisgender populations. Clinicians should consider using TGD patients’ prior weight trajectory, as well as other signs of malnutrition (e.g., heart rate, blood pressure, and laboratory evaluation) when assessing ED/DEB severity and progress with treatment

## Data Availability

Not applicable.
